# Coincidence of cardiovascular disease and breast cancer in females: Multi-modal strategies by predictive, preventive and personalised medicine

**DOI:** 10.1186/1878-5085-5-S1-A87

**Published:** 2014-02-11

**Authors:** Olga Golubnitschaja, Vincenzo Costigliola, Hiroyasu Iso

**Affiliations:** 1 Molecular Diagnostics, Radiological Clinic, Medical Faculty, Friedrich-Wilhelms-University of Bonn, Germany; 2 European Association for Predictive, Preventive and Personalised Medicine, Brussels, Belgium, Avenue des Volontaires, 19, 1160 Brussels, Belgium; 3 Osaka University Graduate School of Medicine, Osaka, Japan

## Epidemiologic background

Regarding most frequent pathologies in females, the causal link between diabetes mellitus and development of severe cardiovascular complications is well acknowledged. In contrast, the co-incidence of the cardiovascular disease (CVD) and breast cancer is poorly understood. This is an extremely unlucky situation, since compared to all other pathologies, both - CVD and breast cancer demonstrate the highest frequency of incidence in females. Hence, the probability to identify a female postmenopausal patient above 55 years old diagnosed with sporadic invasive breast cancer but CVD-free is rather low. However, an obvious coincidence of CVD and breast cancer in peri- and post-menopausal females, synergic co-prevention, common diagnostic tools and multi-drug co-treatments are currently not considered for medical services.

## Current deficits in prevention, diagnosis and treatments of a coincident breast cancer

• Worldwide 8-12 women from hundred get diseased on breast cancer. Around 50% of DCIS (*ductal carcinoma in situ*) will progress to invasive and metastasis disease if untreated, with 20% of recurring ones despite “appropriate” treatments.

• Breast cancer metastatic disease (BCMD) is currently incurable [1].

• Causative factors such as co-incidental ones underlying recurrence of DCIS or progression to invasive disease are currently not known.

• 95-98% are sporadic breast cancer cases *versus* 2-5% familial ones: the majority of sporadic breast cancer cases are peri- and post-menopausal women – the group at significantly higher risk of CVD.

• Co-morbidities in breast cancer are considered rather from view point of psychological aspects and palliative care but not as the realistic model in biomedical research, common diagnostic tools and treatment multimodalities.

• Consistent data do not exist to recognise co-incident pathologies by patient profiling.

• Cost-effective healthcare promotion in breast cancer and its common or even persistent co-morbidities such as CVD and diabetes is not provided.

• A gap between current research performance and clinically applicable results is dramatic.

## ‘Stress proteome’ targets: innovative biomedical approaches meet personalised healthcare

Stress response is the key ability of each organism that enables it to adapt and survive. Individual stress response can explain the individual reactions towards similar conditions and underlies the molecular (pathological) mechanisms of individual outcomes [2]. The minimal stress proteome mainly consists of the following functional groups: Nucleic acid metabolism, Molecular chaperones, DNA damage sensing/repair and cell cycle control, Basic transcription, DNA replication and RNA processing, Translation regulation, protein synthesis and degradation, Redox regulation, CoA metabolism, Energy metabolism and ATPases/ABC transporters [3]. These functional groups represent potential targets to be utilised for complex diagnostic tools and treatments tailored to the person. As reviewed previously [4], genotoxicity is the most fatal consequence of any kind of stress factor, such as the most ubiquitous emotion stress, smoking and alcohol consumption, hyperhomocysteinemia, folate and taurine deficiency, UV and ionising irradiation, fluctuating blood glucose concentration (starvation and diabetes mellitus), asphyxia / ischaemia / stroke / reperfusion, genetic syndromes such as Down’s syndrome, and neurodegenerative diseases such as Huntington’s, Alzheimer’s and Parkinson’s disease, and glaucoma. Recent studies showed individual stress responses to be well or less adapted to life situations. Inadequate stress response can be caused by the dysfunction of red-ox control, cell cycle machinery, apoptotic/repair mechanisms leading to severe pathologies such as human genetic syndromes, accelerated ageing, cardiovascular and neuro/degenerative diseases and cancer [4].

## Multi-modal strategies by predictive, preventive and personalised medicine

➣ Creation of gender-specific profiles for CVD-diagnostics and treatments by well-funded large-scale studies with parity of both sexes including “omics”-based blood analysis, molecular imaging and elaboration of gender-specific questionnaires for accurate description of prodromal and acute symptoms of CVD 5.

➣ Consideration of CVD-specific sex-determinants such as mutated maternal mitochondrial DNA that emerges complications and fatal outcomes of CVD.

➣ Performance of large-scale population studies to identify most frequent profiles of co-incident pathologies for effective treatments suitable for corresponding combination of co-morbidities such as depression/diabetes/CVD/breast cancer in female patients.

➣ Creation of strictly individual treatment priorities and regiments request personalised medical approaches of high plasticity and new generation of multi-cocktail drug compositions.

➣ New drug delivery systems that allow for targeted treatments.

➣ Reasonable convergence between diagnostic and pharmaceutical industry in development of predictive/prognostic approaches based on new multifunctional targets for diagnostic and treatment purposes.

➣ Besides general prevention in populations, there is an urgent need for targeted prevention in childhood, adolescence and adulthood as the most cost-effective approach that requests complete reconsideration of current diagnostic and treatment programmes offered in healthcare systems: major CVD risk factors overweight, obesity and diabetes type 2 are the most frequent nutritional disorders in industrialised countries.

➣ Creation of new programmes in healthcare systems for effective promotion of and financial motivation for healthy life-style.

➣ Creation of the network among healthcare personals dedicated to the task, namely research groups in women’s health (predictive medicine), primary healthcare (preventive measures) and networked clinicians for personalised treatments in female patient cohorts.
